# Health Status of Patients With β-Thalassemia in the West Bank: A Retrospective-Cohort Study

**DOI:** 10.3389/fmed.2021.788758

**Published:** 2021-12-20

**Authors:** Reem Aldwaik, Tamara Abu Mohor, Israa Idyabi, Salam Warasna, Shatha Abdeen, Bashar Karmi, Rania Abu Seir

**Affiliations:** ^1^Department of Medical Laboratory Sciences, Al-Quds University, Jerusalem, Palestine; ^2^Thalassemia Patients' Friends Society, Ramallah, Palestine

**Keywords:** β-thalassemia (β-thal), blood transfusion—adverse effects, iron overload, chelation therapy, thalassemia management

## Abstract

Management of β-thalassemia in developing countries is demanding in the absence of available therapies rather than recurrent transfusions. This study describes the characteristics and evaluates the hematological, biochemical, and hormonal findings of patients with β-thalassemia in the West Bank. We conducted a retrospective cohort study between January 2017 and December 2018. Data were collected through medical files of the patients with β-thalassemia from eight primary healthcare clinics, nine emergency departments, and 11 governmental hospitals across the West Bank. Results of the hematological, biochemical, and hormonal evaluations, in addition to demographic data and the use of iron chelation were included in the study and analyzed. A total of 309 patients with β-thalassemia were included with a male-to-female ratio of 1:1 and an average age of 23.4 ± 10.4 years. The anemic presentation was reported in 78.6% of the patients as indicated by hemoglobin level (mean ± SD = 8.4 ± 1.4 g/dl), and 73.1% had iron overload with serum ferritin (SF) levels ≥ 1,000 μg/L (mean ± SD = 317.8 ± 3,378.8 μg/L). Evaluation of the liver function tests showed that alanine transaminase (ALT) and aspartate transaminase (AST) levels were high among 38.1 and 61.2% of the patients, respectively. ALT and AST showed significant positive correlations with SF levels, while the kidney tests did not. As for iron chelation medications, patients receiving deferoxamine (26.5%) showed significantly higher SF levels compared with patients receiving deferasirox (73.5%). This study highlights the importance of establishing patient-tailored comprehensive assessment and follow-up protocols for the management of β-thalassemia with an emphasis on blood transfusion and iron chelation practices.

## Introduction

β-thalassemia is a recessively autosomal inherited blood disorder characterized by anomalies in the production of the hemoglobin (Hb) beta chain resulting in variable degrees of hemolysis, chronic anemia, and ineffective erythropoiesis (1, 2). The broad spectrum of clinical picture of the patients ranges from the silent asymptomatic state to the lifelong transfusion-dependent anemic state, with its related complications (3). The main treatment option for most patients is supportive care consisting of blood transfusion and iron chelation therapy. Blood transfusion is used to reduce anemia complications, while chelation reduces iron overload caused by chronic blood transfusions. Nevertheless, despite the significant improvements in the management of β-thalassemia, it remains a challenge, especially in low-resource countries, where the burden of thalassemia is the highest (2).

**Figure 1:**
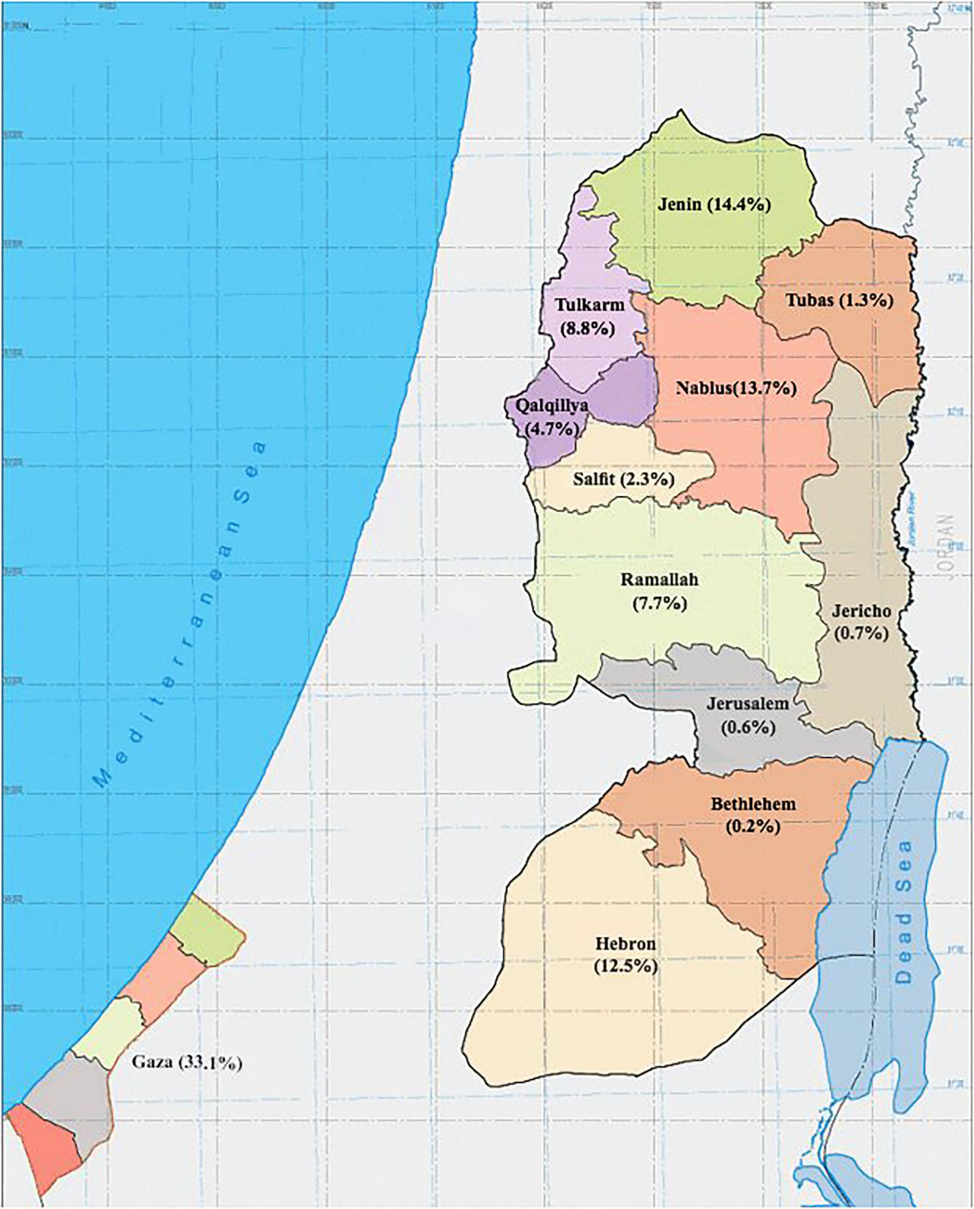
Map of Palestine showing the geographical distribution of patients with β-thalassemia registered in the Thalassemia Patients' Friends Society (TPFS), 2018.

In Palestine, it was estimated that the prevalence of thalassemia carriers in Palestine was around 4% (4). Records from Thalassemia Patients' Friends Society (TPFS) showed that the prevalence of thalassemia was 17.4 per 100,000 population in 2018, with a total number of symptomatic thalassemia patients of 847 including both the West Bank and Gaza Strip. In addition, records have shown that two-thirds of the Palestinian patients were from the West Bank. Males and females had similar disease rates, and 73% of them were between 10 and 30 years old. Furthermore, among patients from the West Bank, the largest proportion was from the northern governorates (47%), while only 9% were from the central governorates, and 12% were from the south (Figure 1). TPFS data show that even though bone marrow transplantation is the only available curative therapy, only 4% of patients with thalassemia in Palestine underwent it (5).

Like the other developing countries, β-thalassemia is a growing health and economic problem. Consequently, significant efforts must be invested in improving the medical care of thalassemia patients to prolong and improve the quality of their life. In this study, we aim to describe the characteristics and evaluate the hematological, biochemical, and hormonal findings of patients with β-thalassemia in the West Bank, in addition to investigating the correlation between serum ferritin (SF) level, iron chelation therapy, and these parameters in order to reflect on the disease management.

## Materials and Methods

A retrospective cohort study was conducted between January 2017 and December 2018. The study utilized data from the medical files of the Ministry of Health (MOH) for 309 patients with β-thalassemia covering eight primary healthcare clinics, nine emergency departments, and 11 governmental hospitals across the West Bank. Inclusion criteria of patients in this study included diagnosis with β-thalassemia major or intermedia and receiving regular or occasional blood transfusion during the study period.

The retrieved data included the demographic characteristics of the patients, laboratory test results, and iron chelation treatment. Demographic characteristics included age, sex, and place of the treatment. Results of laboratory tests included hematological tests (Hb); biochemical tests (serum creatinine, blood urea nitrogen (BUN), alanine aminotransferase (ALT), aspartate aminotransferase (AST), serum total calcium, and SF); and hormonal tests [thyroid-stimulating hormone (TSH), total triiodothyronine (TT3), free thyroxine (FT4), and parathyroid hormone (PTH)]. Test results were categorized as normal or high according to the reference ranges reported in the Tietz Fundamentals of Clinical Chemistry and Molecular Diagnostics (6). SF and Hb levels were categorized according to guidelines of the Thalassemia International Federation (TIF) for the management of transfusion-dependent thalassemia (TDT) (7). We also retrieved data regarding the intake of iron chelation therapy. The available iron chelators in MOH are deferoxamine (DFO) and deferasirox (DFX).

The data were arranged, coded, and analyzed using the IBM Statistical Package of Social Sciences (IBM SPSS Statistics for Windows, Version 25.0. Armonk, NY: IBM Corp.). Frequencies and percentages were calculated for the categorical variables. The continuous measures were presented as means, SD, and ranges. An independent sample *t*-test was used to measure the significance of differences between DFO and DFX receivers in terms of Hb, SF, BUN, serum creatinine, ALT, and AST. Furthermore, Pearson correlation coefficients were calculated to measure the association between Hb, SF, BUN, serum creatinine, ALT, AST, and SF levels. A *p* < 0.05 was considered statistically significant.

The study was approved by the Research Ethics Committee at Al-Quds University and the Palestinian MOH under the reference number 162/1075/2019. The data were previously anonymized and no private information were collected as part of this study; therefore, informed consent from patients was waived for this study.

## Results

A total of 309 patients with β-thalassemia were included in this study with a male-to-female ratio of 1:1 and an average age of 23.4 ± 10.4 years ranging from 2 to 68 years. Table 1 shows the baseline characteristics of patients with β-thalassemia. Most of the patients were from the northern West Bank governorates (63.8%), while 26.2% were from the southern governorates. As for treatment with iron chelation therapy, 72.8% of the patients were under chelation therapy; 77.8% of those patients received DFX, while 28% received DFO (Table 1).

**Table 1:** Demographic characteristics and iron chelation therapy status of β-thalassemia patients.

**Variable**	**Category**	**Frequency (%)**
Sex	Male	154 (49.8)
	Female	155 (50.2)
Age (years)	0–9	20 (8.2)
	10–19	69 (28.3)
	20–29	110 (45.0)
	30–39	28 (11.5)
	≥ 40	17 (7.0)
Region^a^	North	197 (63.8)
	Middle	31 (10.0)
	South	81 (26.2)
Chelation status	Receiver	225 (72.8)
	Non-receiver	84 (27.2)
Type of iron chelator	Deferasirox	175 (77.8)
	Deferoxamine	63 (28.0)

a *North: Jenin, Tubas, Tulkarm, Nablus, Qalqiliya and Salfit; Middle: Ramallah and Jericho; South: Bethlehem and Hebron*.

Table 2 demonstrates the hematological, biochemical, and hormonal baseline characteristics of patients with β-thalassemia. Among the enrolled patients, the mean SF level was 3,175.8 ± 3,378.8 μg/L, ranging from 75.5 to 17,450.4 μg/L. The majority of the patients (73.1%) had SF levels ≥ 1,000 μg/L. Among these patients, 54.6% had SF levels > 2,500 μg/L. The mean Hb level was 8.4 ± 1.4 g/dL. The majority of the patients (78.6%) showed anemic presentation with Hb levels ≤ 9 g/dL. The mean serum creatinine was 44.2 ± 17.7 (μmol/L), whereas the mean of BUN was 5.1 ± 2.4 mmol/L. In addition, we found that 99.2% of the patients had a serum creatinine level within the normal reference range, while 18.2% of the patients had high BUN levels. Evaluating liver function using test results of ALT and AST, elevated ALT levels were observed in 38.1% of the patients, and elevated AST levels were observed among 61.2%. The number of patients who had test results for serum total calcium, PTH, TT3, FT4, and TSH during the 2 years of the study was insufficient to provide a reliable estimation of the hormonal abnormalities or perform any further comparisons.

**Table 2:** The biochemical, hematological, and hormonal baseline characteristics of β-thalassemia patients.

**Indicator**	**Number of patients (n)**	**Category**	**Frequency *n* (%)**	**Mean ± SD**	**Range**
Hemoglobin (g/dL)	285	<6	2 (0.8)	8.4 ± 1.4	5.7–16.8
		6–9	200 (77.8)		
		>9	55 (21.4)		
Serum ferritin (μg/L)	266	250– <1,000	68 (26.9)	3,175.8 ± 3,378.8	75.5–17,450.0
		1,000–2,500	84 (33.2)		
		>2,500	101 (39.9)		
Serum creatinine (μmol/L)	277	Normal	253 (99.2)	44.2 ± 17.7	17.7–123.8
		High	2 (0.8)		
BUN^a^ (mmol/L)	269	Low	5 (2.0)	5.1 ± 2.4	1.3–16.2
		Normal	197 (79.8)		
		High	45 (18.2)		
ALT^b^ (IU/L)	257	Normal	148 (61.9)	41.8 ± 36.5	5.0–208.3
		High	91 (38.1)		
AST^c^ (IU/L)	255	Normal	92 (38.8)	52.0 ± 40.6	11.2–369.0
		High	145 (61.2)		
Serum total calcium (mmol/L)	65	Low	9 (14.0)	2.3 ± 0.3	1.4–2.6
		Normal	54 (84.4)		
		High	1 (1.6)		
PTH^d^ (pmol/L)	53	Low	4 (8.0)	620.9 ± 440.6	20.0–28,00.7
		Normal	25 (50.0)		
		High	21 (42.0)		
TT3^e^ (nmol/L)	71	Normal	47 (66.2)	2.9 ± 1.4	1.1–5.9
		High	24 (33.8)		
FT4^f^ (pmol/L)	80	Low	3 (3.8)	3.7 ± 3.7	0.5–13.8
		Normal	50 (63.3)		
		High	26 (32.9)		
TSH^g^ (mIU/L)	165	Low	1 (0.7)	3.0 ± 1.6	0.1–9.3
		Normal	133 (84.7)		
		High	23 (14.6)		

a *BUN, blood urea nitrogen*;

b *ALT, alanine transaminase*;

c *AST, aspartate transaminase*;

d *PTH, parathyroid hormone*;

e *TT3, total triiodothyronine*;

f *FT4, free thyroxine*;

g *TSH, thyroid-stimulating hormone*.

Classification of the hematological, biochemical, and hormonal baseline characteristics of patients with β-thalassemia by age showed that while patients between the ages of 20 and 29 years had the highest SF levels, most patients in all the age groups had an anemic presentation with Hb levels ≤ 9 g/dL. Furthermore, abnormal laboratory findings were the most commonly observed among older patients; however, this was not true in patients ≥40 years old (Table 3).

**Table 3:** The biochemical, hematological, and hormonal baseline characteristics of patients with β-thalassemia by age.

**Indicator**	**Category**	**Age group (years)** ***n*** **(%)**				
		**0–9**	**10–19**	**20–29**	**30–39**	**≥40**
Hemoglobin (g/dL)	<6	0 (0.0)	0 (0.0)	2 (2.1)	0 (0.0)	0 (0.0)
	6–9	16 (80.0)	55 (83.3)	72 (74.2)	22 (81.5)	14 (82.4)
	>9	4 (20.0)	11 (16.7)	23 (23.7)	5 (18.5)	3 (17.6)
Serum ferritin (μg/L)	250– <1,000	6 (33.3)	15 (24.6)	21 (23.3)	5 (22.7)	5 (31.3)
	1,000–2,500	6 (33.3)	23 (37.7)	23 (25.6)	13 (59.1)	5 (31.3)
	>2,500	6 (33.3)	23 (37.7)	46 (51.1)	4 (18.2)	6 (37.4)
Serum creatinine (μmol/L)	Normal	16 (100.0)	62 (100.0)	83 (100.0)	19 (95.0)	15 (93.8)
	High	0 (0.0)	0 (0.0)	0 (0.0)	1 (5.0)	1 (6.2)
BUN^a^ (mmol/L)	Low	1 (5.9)	1 (1.7)	1 (1.2)	0 (0.0)	1 (6.2)
	Normal	14 (82.3)	49 (84.5)	61 (74.4)	14 (70.0)	12 (75.0)
	High	2 (11.8)	8 (13.8)	20 (24.4)	6 (30.0)	3 (18.8)
ALT^b^ (IU/L)	Normal	10 (58.8)	40 (67.8)	38 (49.4)	14 (82.4)	10 (62.5)
	High	7 (41.2)	19 (32.2)	39 (50.6)	3 (17.6)	6 (37.5)
AST^c^ (IU/L)	Normal	4 (23.5)	24 (40.7)	28 (36.4)	8 (47.1)	6 (40.0)
	High	13 (76.5)	35 (59.3)	49 (63.6)	9 (52.9)	9 (60.0)
Serum total calcium (mmol/L)	Low	0 (0.0)	2 (12.5)	4 (19.0)	1 (12.5)	1 (33.3)
	Normal	4 (80.0)	14 (87.5)	17 (81.0)	7 (87.5)	2 (66.7)
	High	1 (20.0)	0 (0.0)	0 (0.0)	0 (0.0)	0 (0.0)
PTH^d^ (pmol/L)	Low	0 (0.0)	0 (0.0)	2 (14.1)	1 (50.0)	1 (50.0)
	Normal	2 (100.0)	9 (64.3)	6 (42.9)	1 (50.0)	0 (0.0)
	High	0 (0.0)	5 (35.7)	6 (42.9)	0 (0.0)	1 (50.0)
TT3^e^ (nmol/L)	Normal	3 (100.0)	11 (73.3)	17 (81.0)	2 (66.7)	2 (100.0)
	High	0 (0.0)	4 (26.7)	4 (19.0)	1 (33.3)	0 (0.0)
FT4^f^ (pmol/L)	Low	0 (0.0)	1 (5.3)	1 (4.2)	0 (0.0)	1 (50.0)
	Normal	4 (100.0)	17 (89.4)	17 (70.8)	5 (100.0)	1 (50.0)
	High	0 (0.0)	1 (5.3)	6 (25.0)	0 (0.0)	0 (0.0)
TSH^g^ (mIU/L)	Low	0 (0.0)	0 (0.0)	0 (0.0)	0 (0.0)	0 (0.0)
	Normal	8 (80.0)	32 (86.5)	44 (84.6)	11 (78.6)	10 (90.9)
	High	2 (20.0)	5 (13.5)	8 (15.4)	3 (21.4)	1 (9.1)

a *BUN, blood urea nitrogen*;

b *ALT, alanine transaminase*;

c *AST, aspartate transaminase*;

d *PTH, parathyroid hormone*;

e *TT3, total triiodothyronine*;

f *FT4, free thyroxine*;

g *TSH, thyroid-stimulating hormone*.

Table 4 demonstrates a comparison in the means of the hematological and biochemical parameters by the type of chelation therapy (DFX vs. DFO). The mean SF level of DFO receivers (6,272.8 ± 5,781.3 μg/L) was significantly higher than the mean SF level of DFX receivers (2,965.9 ± 2,755.8 μg/L), with a *p* = 0.009. Furthermore, the comparison between males and females in SF levels showed no significant statistical difference (*p* = 0.973).

**Table 4:** Means and SDs of all hematological and biochemical parameters for deferasirox receivers and deferoxamine receivers.

**Indicator**	**Deferasirox (DFX)**	**Deferoxamine (DFO)**	***P*-value**
Serum ferritin (μg/L)	2,965.9 ± 2,755.8	6,272.8 ± 5,781.3	0.009^*^
Hemoglobin (g/dL)	8.1 ± 0.8	8.4 ± 1.2	0.152
BUN^a^ (mmol/L)	5.7 ± 2.5	4.3 ± 0.04	0.597
ALT^b^ (IU/L)	46.1 ± 35.6	55.3 ± 32.6	0.289
AST^c^ (IU/L)	52.9 ± 30.3	71.8 ± 77.8	0.311
Serum creatinine (μmol/L)	35.4 ± 8.8	44.2 ± 8.8	0.597

a *BUN, blood urea nitrogen*;

b *ALT, alanine transaminase*;

c *AST, aspartate transaminase*.

* *P <0.05*.

Finally, we measured the correlation between hematological and biochemical parameters and SF levels using Pearson correlation. We found a significant positive correlation between SF and ALT (*r* = 0.527, *p* < 0.0001) and SF and AST (*r* = 0.254, *p* < 0.0001). On the contrary, we did not find any significant correlations between serum creatinine, BUN, Hb, and SF levels (*p* = 0.553, 0.280, and 0.057, respectively) (Table 5).

**Table 5:** The correlation between serum ferritin levels and all the hematological and biochemical parameters of patients with β-thalassemia.

**Indicator**	**Correlation coefficient (r)**	***P*-value**
Hemoglobin (g/dL)	−0.125	0.057
BUN^a^ (mmol/L)	−0.072	0.280
ALT^b^ (IU/L)	0.527	<0.0001^*^
AST^c^ (IU/L)	0.254	<0.0001^*^
Serum creatinine (μmol/L)	−0.039	0.553

a *BUN, blood urea nitrogen*;

b *ALT, alanine transaminase*;

c *AST, aspartate transaminase*.

* *P <0.05*.

## Discussion

Managing β-thalassemia syndrome in the developing countries poses a major challenge to the healthcare system and a burden on the public health resources (8). Therefore, understanding the current situation should be a high priority, particularly with an emphasis on blood transfusion therapy, iron chelation therapy, and the management of associated comorbidities. Previously, a cross-sectional study conducted in Gaza Strip revealed deteriorated hematological and biochemical statuses of patients with β-thalassemia (9). This study assessed the health status of patients with β-thalassemia in the West Bank with a representative sample size in terms of age, sex, and geographical distribution. The outcome of this evaluation study could be used to modify the existing policies in the management of patients with β-thalassemia, emphasizing blood transfusion, and iron chelation therapy practices in Palestine. In addition, our findings could roadmap the bridge between researchers and healthcare professionals to ensure the best potential patient outcomes.

The advent of safe blood transfusion with the adjuvant of the chelation therapy has significantly increased life expectancy of the patients, with the close monitoring and following-up programs of the patients with β-thalassemia can now survive into the fourth and fifth decades of their lives (10, 11). In this study, a total of 309 patients with β-thalassemia representing the West Bank were included. Comparing our sample demographics to other areas, the average age of patients with β-thalassemia in the neighborhood countries such as Lebanon was similar to ours (23.4 ± 10.4 years in the West Bank compared with 21.0 ± 11.0 years in Lebanon) (12). On the contrary, a better life expectancy was reported in Taiwan, with an average age of 37.8 ± 23.7 years (10). Furthermore, the high burden of β-thalassemia in the West Bank, especially in the north, could be explained by the preference for traditional consanguineous marriages and the large family sizes associated with social and cultural considerations (13, 14).

Blood transfusion is lifesaving for patients with β-thalassemia. The main goal of blood transfusion is the correction of anemia, and the decision to start transfusion therapy should be mainly based on the assessment of Hb levels (2). For accurate monitoring of the effectiveness of transfusion therapy, continuous follow-up is required at each transfusion for pre- and post-transfusion Hb, hematocrit of the blood unit, daily Hb fall, and the transfusional interval (2). According to recommendations of TIF, the current acceptable mean Hb is 12 g/dl with a post-transfusion Hb of 14–15 g/dl and a pretransfusion Hb of 9–10.5 g/dl (7). Among the patients in our study, an anemic presentation was widely observed with a mean Hb during the 2-year follow-up of 8.4 ± 1.4 g/dl. These low levels of Hb indicate poor management of transfusion treatment regimens, lack of adherence to international guidelines, and possibly failure to achieve a regular adherence to the treatment by patients. Similar to our findings, low Hb levels were seen among patients with β-thalassemia in Egypt (8.2 ± 1.8 g/dl) (15), Jordan (8.9 ± 2.4 g/dl) (16), and Pakistan (7.7 ± 0.1 g/dl) (17).

Even though blood transfusion is lifesaving for patients with β-thalassemia, it burdens the body with excess iron that ultimately results in hemosiderosis and other related comorbidities leading to consequential and irrevocable biological damages (9, 18). Based on Guidelines for managing TDT of TIF, monitoring body iron levels by measuring liver iron concentration through MRI-based methods is recommended to maintain adequate and safe management (7). However, MRI-based methods are not yet available in the West Bank. On the other hand, serial measurement of SF concentration is the most commonly reliable non-invasive method used to monitor iron load. Therefore, it is used as a reasonable estimate of the total body iron. However, as a single value, SF could be influenced by factors such as liver disease, inflammatory disorders, and cancer, and it is not a good indicator of myocardial iron overload (2, 19). Nevertheless, SF might be the only feasible test for measuring iron burden and efficacy of chelation therapy in the developing countries (20).

In this study, the mean SF level of our patients with β-thalassemia was markedly higher than the cutoff point recommended by the Guidelines of TIF for the management of TDT (7), which must be maintained below 1,000 μg/L (3,175.8 ± 3,378.8 μg/L). In addition, SF did not vary significantly in terms of sex. Similar to our findings, high SF levels were reported among Pakistani patients with thalassemia 3,087.6 ± 1,625.0 μg/L (21). In contrast, in the developed countries such as France, better SF levels were reported among their patients with β-thalassemia (1,240 μg/L) (22). High SF levels indicate improper or unmonitored blood transfusion and/or therapeutic chelation practices (23, 24).

With the support of blood transfusion, iron chelation therapy is the best feasible choice to decrease comorbidities and increase life expectancy of the patients (25). Worldwide, the available iron chelators are DFO, DFX, and DFP. In the West Bank, only DFO and DFX are included in the Essential Drug List; however, availability of drugs in MOH is such a dilemma due to the high cost; therefore, these drugs are usually provided as donations from other countries. As a result, drug availability is an important factor affecting the choice of drug of the clinicians and the provision of the prescribed medication for the patients on time to use (26). In this study, we found that most of the patients (72.8%) received chelation therapy during the study period. Among those, 77.8% were prescribed DFX and 28% were prescribed DFO. Comparison of SF levels among our patients revealed that the mean SF level for DFO receivers (6,272.8 ± 5,781.3 μg/L) was significantly higher than the mean SF level for DFX receivers (2,965.9 ± 2,755.8 μg/L).

In comparison, DFO is the standard iron chelator in Gaza, and dramatically higher SF levels were reported 7,115.6 ± 3,561.7 μg/L (9). Studies have shown that DFO, administered subcutaneously or intravenously, has a short half-life. In addition, DFO has the lower efficacy with a low-compliance rate because of the dose-related toxicity and the associated discomfort during administration (20, 27). DFX has a favorable safety profile since it is an oral suspension with less toxicity and higher compliance and efficacy compared to DFO, but it is an expensive chelator (28). Experimental trials showed that using DFX and DFO combined results in excreting more iron without any noticeable significant complications (29). Thus, this approach can be promising, especially in the poor-developing countries with the limited resources such as Palestine. However, further research is required to understand the factors affecting response to iron chelation therapy, including indications for starting and adjusting treatment regimen, dosage, drug availability, and compliance of the patient.

In transfusion-dependent patients with β-thalassemia, the liver is the first affected organ by the toxic effect of excess iron on hepatocytes. The prevalence of the impaired liver function is estimated at 40.5% (30, 31). In our study, liver dysfunction was manifested by the elevated levels of ALT and AST among our patients. This elevation was significantly correlated with an increase in SF levels. A previous study conducted in India reported similar findings. The study showed that once SF levels increase above 1,000 ng/ml and the total number of blood transfusions goes beyond 30 times, the liver impairment starts (30). Therefore, monitoring and evaluating hepatic iron overload is vital to protect the liver and the rest of the body (32). In addition to iron overload, elevation in the levels of liver enzymes in both the transfusion-dependent and transfusion-independent patients with β-thalassemia might be attributed to other factors such as viral infection (hepatitis B and C viruses), hepatic siderosis, bile obstruction, portal fibrosis, and even cirrhosis (30).

The renal dysfunctions, including the renal tubular and glomerular injuries were reported to be the fourth most common morbidity of β-thalassemia (4%) (31). Several studies suggested the involvement of iron chelators in the pathophysiology of the renal disease among patients with thalassemia (31). In our study, most of the enrolled patients had normal serum creatinine levels, and only 18.2% of them had a slight increase in BUN levels. In addition, there was no statistically significant association between SF levels and BUN or serum creatinine. Similarly, the reported BUN and serum creatinine levels were within the normal ranges in a study conducted in Iran, indicating that the function of the renal tubular and glomerular was not adversely affected by iron overload (33).

Endocrinopathies are the most common complications of β-thalassemia syndrome and iron overload, with a prevalence of 44.7% (34). Endocrinopathies could result in the delayed puberty and hypogonadism (40.5%), short stature and retarded growth (30.8%), impaired glucose tolerance, and diabetes mellitus (9.9%), hypoparathyroidism (6.9%), hypothyroidism (3.2%), and adrenal insufficiency (7). Despite the high rates of endocrinopathies and the broad spectrum of associated comorbidities, we found that a small proportion of patients with β-thalassemia in West Bank had their endocrine functions evaluated. In addition, among the patients who underwent testing for endocrinopathies, the performed tests were not enough to make an interpretation or to establish any diagnosis. Although this study did not provide enough data to estimate the burden of endocrinopathies among patients in West Bank, the absence of regular endocrine assessment and follow-up was highlighted as one of the significant gaps in the management of β-thalassemia. The continuous evaluation of the toxic effect of the iron overload on endocrinopathies could be accomplished through regular monitoring of signs and symptoms of endocrine complications. Together with the appropriate iron chelation practice, continuous monitoring could ensure early diagnosis, prevention, and timely treatment of such complications (7, 35).

Classification of the hematological, biochemical, and hormonal baseline characteristics of patients with β-thalassemia by age showed that the older patients generally had a higher prevalence of complications and worse profiles. However, this was not true among patients ≥40 years old, which could be because patients with less severe phenotypes of thalassemia survive longer. Furthermore, poor management of transfusion therapy and persistent anemia at the young ages would affect growth of children, resulting in the worst disease outcomes later in the life.

In summary, this study showed that regardless of the provision of blood transfusion services and iron chelation therapies, the lack of standardized protocols for the management of thalassemia is a major problem in West Bank. For the majority of the patients, Hb levels were far below the international recommendations, and uncontrolled SF levels were found, reflecting the absence of evidence-based administration of blood transfusion and iron chelation therapy. Furthermore, the data indicated improper assessment and follow-up for comorbidities associated with iron overload. It is also important to remember that blood transfusion is associated with other adverse outcomes such as transfusion reactions; the development of allo- and autoantibodies, which can further complicate thalassemia management; and blood-borne infectious diseases such as hepatitis and HIV.

The limitations of this study were mainly derived from the lack of adequate care. Furthermore, we did not include patients from Gaza Strip in this study. The management of patients with thalassemia in Gaza Strip is affected by a broader range of factors, including the political and economic situation of the area limiting their access to healthcare and access to testing and treatment. Moreover, a major limitation in this study was the incomplete patient information on the health information system, which is mainly attributed to the fragmentation of the healthcare system. The fragmentation of the healthcare information system poses a serious challenge to managing patients with thalassemia in Palestine.

## Conclusions

In conclusion, these findings highlight the need for the comprehensive assessment protocols and evidence-based practices as they are crucial for the reduction of β-thalassemia-associated morbidity and mortality. Therefore, regular evaluation and follow-up with emphasis on blood transfusion and iron chelation practices are highly recommended to improve clinical picture of the patients, life expectancy, and quality of life. Also, the treatment has to be tailored for each patient due to the wide heterogeneity of β-thalassemia. In addition, despite being the only available curable treatment, stem cell transplantation is not feasible in our country. Thus, establishing advocacy programs aiming to increase adherence of the patients to treatment and follow-up programs is a high priority. This could be achieved through the collaboration of a national multidisciplinary team consisting of the hematologists, endocrinologists, cardiologists, ophthalmologists, dentists, psychologists, social workers, and dietitians. Finally, it is noteworthy to mention that the absence of evidence-based transfusional regimens and follow-up protocols are not the only gaps in the management of patients with thalassemia. Furthermore, although genetic testing of thalassemia is becoming important, especially in complex atypical thalassemias (36), molecular diagnosis is rarely performed. This service is not provided in MOH facilities, which is another gap in managing patients with thalassemia in West Bank.

## Data Availability Statement

The raw data supporting the conclusions of this article will be made available by the authors, without undue reservation.

## Ethics Statement

The studies involving human participants were reviewed and approved by Research Ethics Committee (REC), Al-Quds University, Jerusalem, Palestine. Written informed consent from the participants' legal guardian/next of kin was not required to participate in this study in accordance with the national legislation and the institutional requirements.

## Author Contributions

RAb conceived and designed the study and reviewed the final draft. BK facilitated the process of data collection through the MOH and contributed to confirming the cases selected in the study and interpretation of the results. RAl, TA, II, SA, and SW were responsible for the data collection, entry, analysis, and interpretation. RAl drafted the manuscript. All the authors contributed to manuscript revision, read, and approved the submitted version.

## Conflict of Interest

The authors declare that the research was conducted in the absence of any commercial or financial relationships that could be construed as a potential conflict of interest.

## Publisher's Note

All claims expressed in this article are solely those of the authors and do not necessarily represent those of their affiliated organizations, or those of the publisher, the editors and the reviewers. Any product that may be evaluated in this article, or claim that may be made by its manufacturer, is not guaranteed or endorsed by the publisher.
